# Dependable Sensor Fault Reconstruction in Air-Path System of Heavy-Duty Diesel Engines

**DOI:** 10.3390/s21237788

**Published:** 2021-11-23

**Authors:** Ashkan Taherkhani, Farhad Bayat, Saleh Mobayen, Andrzej Bartoszewicz

**Affiliations:** 1 Department of Electrical Engineering, University of Zanjan, Zanjan 45371-38791, Iran; ashkantaherkhani7@gmail.com; 2 Future Technology Research Center, National Yunlin University of Science and Technology, Yunlin 64002, Taiwan; 3 Institute of Automatic Control, Lodz University of Technology, 90924 Lodz, Poland; andrzej.bartoszewicz@p.lodz.pl

**Keywords:** sensor reliability, linear matrix inequality, robustness, sliding mode observer, diesel engine

## Abstract

This paper addresses the problem of robust sensor faults detection and isolation in the air-path system of heavy-duty diesel engines, which has not been completely considered in the literature. Calibration or the total failure of a sensor can cause sensor faults. In the worst-case scenario, the engines can be totally damaged by the sensor faults. For this purpose, a second-order sliding mode observer is proposed to reconstruct the sensor faults in the presence of unknown external disturbances. To this aim, the concept of the equivalent output error injection method and the linear matrix inequality (LMI) tool are utilized to minimize the effects of uncertainties and disturbances on the reconstructed fault signals. The simulation results verify the performance and robustness of the proposed method. By reconstructing the sensor faults, the whole system can be prevented from failing before the corrupted sensor measurements are used by the controller.

## 1. Introduction

Nowadays, industrial applications need increasingly reliable approaches to operate at a high level of performance. Fault Detection and Isolation (FDI) methods are becoming much more effective at making a process reliable [1]. The sensor faults are the most frequent faults that occur in many control systems such as wind turbines [2], motor drives [3], electro-hydraulic and rotating machines [4,5], power systems and renewable energies [6].

In the presence of unknown signals, the earliest observer (i.e., Luenberger observer [7]) may be unable to force the output estimation error to converge to zero, and consequently the observer states will not converge to system states [8]. In the presence of disturbances and faults, a sliding mode observer (SMO) can be used to minimize the effect of disturbances on fault reconstruction signals [9]. In [10], the faults are reconstructed using a sliding mode observer in a system with no unknown signals. Sensor and actuator faults are reconstructed in [11] using an adaptive sliding mode observer in a 5 MW wind turbine system. The second-order sliding mode observer (SOSMO) is becoming a more interesting method these days [12,13]. Chattering reduction, higher accuracy motion, and finite-time convergence for dynamical systems are three important features of SOSMO [14,15].

Heavy-duty diesel engines are industrial equipment which is generally commercial equipment with GVWR (Gross Vehicle Weight Rating) of 10,000 pounds or more. Diesel engines have several advantages over gasoline engines, like creating an optimal compromise between fuel consumption and given exhaust legislation level by producing the requested torque [16]. Model predictive control in [17,18] and sliding mode control in [19] are two common control strategies in diesel engine air-path systems.

Sensor faults can thoroughly damage the system. With the loss of accuracy and showing a constant value rather than the true value due to the loss of sensitivity of sensors, reconstruction of these faults has currently become more crucial. There are several methods for fault reconstruction in industrial processes. In [20], two design methods were proposed to reconstruct known and unknown faults for a class of nonlinear systems using linear matrix inequality (LMI). In [21], a terminal sliding-mode observer (TSMO) was proposed for reconstructing faults in a class of second-order multi-input and multi-output (MIMO) nonlinear systems. In [22], considering the actuator and sensor faults of Markovian jump systems, a fault reconstruction-based method was developed using two novel observer schemes. In [23], a sliding mode observer was designed for the fault diagnosis problem of a linear time-invariant system. An adaptive super twisting observer was used in [4] for fault reconstruction in electro-hydraulic systems in the presence of uncertainties. Multiple sliding mode observers in the cascade were proposed in [24] for robust fault reconstruction in uncertain linear systems. In [25], a sliding mode observer was suggested for the fault reconstruction problem in a Takagi–Sugeno fuzzy descriptor system. In [26], a higher terminal sliding mode observer was proposed for robust fault reconstruction of the nonlinear Lipschitz system using the LMI concept. To the best of the authors’ knowledge, the investigation of sensor fault in an engine air-path has not been reported yet. This problem is addressed in this paper. The main contributions of this paper are as follows:

1. A diesel engine air-path system is studied completely, and by considering the sensor faults and disturbances which can affect the system, a complete model of the air-path system is presented.
2. The nonlinear discontinuous term causes chattering of fault reconstruction, while proper higher-order sliding mode observer can weaken this problem. A higher-order sliding mode observer can also eliminate the deviation from true states and fault reconstruction in the presence of disturbances. Therefore, in the next step, a second-order sliding mode observer is designed.
3. Although this paper’s approach is developed for a diesel engine air-path system, it can be broadened to other industrial processes and applications for reconstructing various possible faults in the presence of disturbances.

The proposed sensor fault reconstruction method is investigated according to the designed observer, and the simulation results for an air-path of a heavy-duty diesel engine system are compared to a sliding mode observer based approach.

## 2. Diesel Engine Air-Path Modeling

### 2.1. Diesel Engine Overview

**Figure 1:**
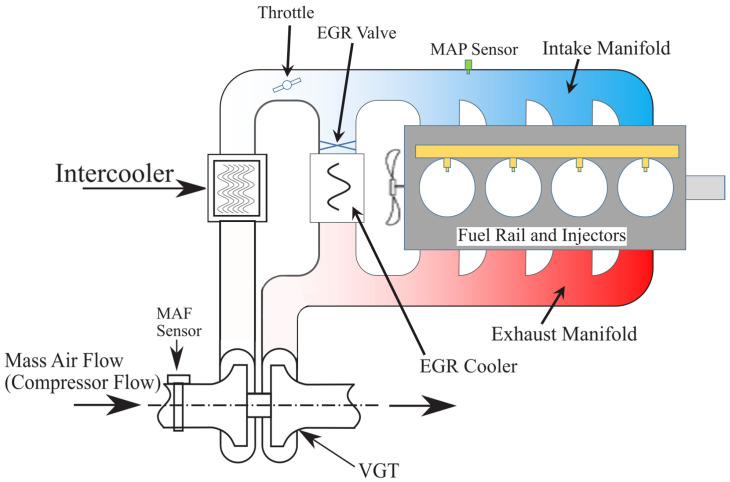
A diesel engine air-path diagram.

Diesel engines are a kind of energy converter which convert fuel energy into mechanical energy. They work according to supply, and heat is released by combustion in an engine forming a thermodynamic cycle [27]. Modeling and details of control methods for diesel engines have been studied in [28,29]. Figure 1 shows the diagram of the diesel engine air-path system.

### 2.2. Manifold Modeling

The dynamics of diesel engine air-path system is modeled as follows:

(1) $$\begin{array}{c} {\dot{p}}_{im}=\frac{R_{a}T_{im}}{V_{im}}\left(W_{air}+W_{egr}-W_{ei}\right) \end{array}$$

where $p_{im}$ is the intake gas pressure and is considered as the system’s manifold. The system has the ideal gas constant $R_{a}$ for the air. $T_{im}$ is the intake gas temperature, which is assumed to be constant, and $V_{im}$ is the intake manifold volume. $W_{air}$, $W_{egr}$ and $W_{ei}$ are the air rate, Exhaust Gas Recirculation (EGR) rate and the cylinder mass flow rate, respectively.

### 2.3. Turbocharger Speed Modeling

According to Newton’s second law,

(2) $$\begin{array}{c} {\dot{\omega }}_{t}=\frac{P_{t}\eta_{m}-P_{c}}{J_{t}\omega_{t}} \end{array}$$

where $\eta_{m}$ is the mechanical efficiency of the turbocharger and $J_{t}$ is the rotating inertia of the turbocharger. $P_{t}$ and $P_{c}$ are the turbine power and the compressor power, respectively, which are calculated as follows:

(3) $$\begin{array}{c} \begin{array}{c} P_{t}=\eta_{tm}W_{t}C_{pe}T_{em}\left(1-{\left(\frac{P_{amb}}{P_{em}}\right)}^{\left(1-\frac{1}{\gamma_{e}}\right)}\right)\\ P_{c}=\frac{1}{\eta_{c}}\left(W_{air}+W_{egr}\right)C_{pa}T_{amb}\left({\left(\frac{P_{im}}{P_{amb}}\right)}^{\left(1-\frac{1}{\gamma_{a}}\right)}-1\right) \end{array} \end{array}$$

where $\eta_{tm}$ is the turbine efficiency, $\eta_{c}$ is the compressor efficiency, $C_{pe}$ is the heat capacity of exhaust gas, $\gamma_{e}$ is the heat capacity ratio of exhaust gas, $C_{pa}$ is the heat capacity of intake gas and $\gamma_{a}$ is the heat capacity ratio of intake gas. $T_{em}$, $T_{amb}$, $P_{em}$ and $P_{amb}$ are also the temperature of the gas in the exhaust before the turbine, the ambient temperature of the intake gas, the exhaust pressure before the turbine, the pressure ratio of the downstream pressure of EGR valve, respectively. By considering density variation in the mass flow, the turbine mass flow $W_{t}$ is modeled as

(4) $$\begin{array}{c} \frac{W_{t}\sqrt{T_{em}R_{a}}}{P_{em}}=A_{vgt}f_{\Pi } \end{array}$$

where $A_{vgt}$ is the effective area that the gas flow through, modeled as [19]

(5) $$\begin{array}{c} A_{vgt}=A_{vgt_{\max}}\left(1-e^{-k_{vgt}u_{vgt}^{c}}\right) \end{array}$$

where $A_{vgt_{\max}}$ is the maximum nominal flow area of the variable-geometry turbocharger (VGT) actuation, and $u_{vgt}^{c}$ and $k_{vgt}$ are the VGT actuator dynamic and a constant value, respectively. $f_{\Pi }$ shows that the mass flow depends on the pressure ratio and is equal to

(6) $$\begin{array}{c} f_{\Pi }=\sqrt{1-{\left(\frac{P_{em_{td}}}{P_{em}}\right)}^{K_{t}}} \end{array}$$

where $P_{em_{td}}$ is the downstream pressure of turbine, and $K_{t}$ is constant.

### 2.4. EGR Mass Flow Modeling

The fluid flows (airflow and exhaust gas flow) through the engine are controlled by the EGR throttle, EGR valve and variable geometry turbocharger (VGT). The EGR-valve mass flow through a variable area is modeled as

(7) $$\begin{array}{c} W_{egr}=\frac{A_{egr}P_{em}}{\sqrt{T_{em}R_{e}}}\psi_{egr} \end{array}$$

where $R_{e}$ is the ideal gas constant for exhaust. $\psi_{egr}$ is a function of the pressure ratio of $P_{amb}$ and $P_{em_{td}}$. $A_{egr}$ is the effective flow area of EGR valve, and is calculated as [19]

(8) $$\begin{array}{c} A_{egr}=A_{egr_{\max}}u_{egr}=A_{egr_{\max}}\left(1-e^{-k_{egr}u_{egr}^{c}}\right) \end{array}$$

where $A_{egr_{\max}}$ is the maximum nominal flow area of EGR actuation, and $u_{egr}^{c}$ and $k_{egr}$ are EGR actuator dynamic and a constant value, respectively. In (1), the term $\left(W_{air}+W_{egr}\right)=W_{c}$ is called compressed air-flow and is modeled as

(9) $$\begin{array}{c} W_{c}=\frac{P_{amb}\pi R_{c}^{3}\omega_{t}\varphi_{c}}{R_{a}T_{amb}} \end{array}$$

where $R_{c}$ and $\varphi_{c}$ are the radius of the compressor blade and volumetric flow coefficient, respectively.

### 2.5. Cylinder Flow Modeling

From the intake manifold to the cylinders, the cylinder mass flow model is obtained as

(10) $$\begin{array}{c} W_{ei}=\frac{\eta_{vol}P_{im}\omega_{e}V_{d}}{120R_{a}T_{im}} \end{array}$$

where $\eta_{vol}$, $\omega_{e}$ and $V_{d}$ are the volumetric efficiency, the engine speed and the displaced volume, respectively.

### 2.6. Unified Model of a Diesel Engine Air-Path

By combining Equations (1)–(9), the state-space model of a diesel engine air-path is obtained as follows:

(11) $$\begin{array}{c} \begin{array}{c} \dot{x}=\underset{A}{\underset{︸}{\left[\begin{array}{ccc} R_{1} & R_{2} & 0\\ 0 & R_{3} & 0\\ 0 & 0 & 0 \end{array}\right]}}x+\underset{B}{\underset{︸}{\left[\begin{array}{cc} 0 & 0\\ R_{4} & 0\\ 0 & R_{5} \end{array}\right]}}u\\ y=\underset{C}{\underset{︸}{\left[\begin{array}{ccc} 1 & 0 & 0\\ 0 & 0 & 1 \end{array}\right]}}x \end{array} \end{array}$$

where

(12) $$\begin{array}{c} \begin{array}{c} R_{1}=\frac{\eta_{vol}V_{d}\omega_{e}}{120V_{im}}\\ R_{2}=\frac{T_{im}P_{amb}\pi R_{c}^{3}\varphi_{c}}{V_{im}T_{amb}}\\ R_{3}=-\frac{P_{amb}\pi R_{c}^{3}\varphi_{c}C_{p_{a}}}{R_{a}\eta_{c}\omega_{t}J_{t}}\left({\left(\frac{P_{im}}{P_{amb}}\right)}^{\left(1-\frac{1}{\gamma_{a}}\right)}-1\right)\\ R_{4}=\frac{A_{vgt_{\max}}P_{em}f_{\Pi }\eta_{tm}C_{pe}T_{em}}{\omega_{t}J_{t}\sqrt{T_{em}R_{e}}}\left(1-{\left(\frac{P_{amb}}{P_{em}}\right)}^{\left(1-\frac{1}{\gamma_{e}}\right)}\right)\\ R_{5}=\frac{A_{egr_{\max}}P_{em}}{\sqrt{T_{em}R_{e}}}\psi_{egr} \end{array} \end{array}$$

In (11), the state vector and the control input vector are denoted as $x={\left[\begin{array}{ccc} P_{im} & \omega_{t} & W_{egr} \end{array}\right]}^{T}$ and $u={\left[\begin{array}{cc} u_{vgt} & du_{egr} \end{array}\right]}^{T}$, respectively, where $u_{vgt}$ and $u_{egr}$ are the VGT and EGR normalized actuator signals.

### 2.7. Disturbance and Sensor Fault Modeling

In this system, the measured disturbance is the engine speed ($\omega_{e}$). The faults in the manifold air pressure sensor, which is used to measure the intake gas pressure, and EGR mass flow rate sensor are considered. The EGR mass flow rate sensor is located in the path of the EGR valve and the intake manifold in Figure 1. Note that the EGR mass flow rate is estimated by the measured pressures on both sides of the EGR system. Accordingly, from (11), the output state-space model will be written as

(13) $$\begin{array}{c} y=\left[\begin{array}{ccc} 1 & 0 & 0\\ 0 & 0 & 1 \end{array}\right]\left[\begin{array}{c} P_{im}\\ \omega_{t}\\ W_{egr} \end{array}\right]+F\left[\begin{array}{c} f_{P_{im}}\\ f_{W_{egr}} \end{array}\right] \end{array}$$

where $f_{P_{im}}$ and $f_{W_{egr}}$ are the manifold gas pressure sensor fault and the EGR mass flow rate sensor fault, respectively.

Finally, the model of diesel engine air-path system in the presence of external disturbance and sensor faults is obtained as

(14) $$\begin{array}{c} \begin{array}{c} \dot{x}\left(t\right)=Ax\left(t\right)+Bu\left(t\right)+D\omega_{e}\\ y(t)=Cx(t)+Ff(t) \end{array} \end{array}$$

where $A\in R^{n\times n}$, $B\in R^{n\times m}$, $D\in R^{n\times q}$, $C\in R^{p\times n}$ and $F\in R^{p\times l}$ are the distribution matrices of state, control input, disturbance, output and sensor fault, respectively.

Consider a new state $z_{f}\left(t\right)$ that filters the output y(t) given by

(15) $$\begin{array}{c} {\dot{z}}_{f}\left(t\right)=-A_{f}z_{f}\left(t\right)+A_{f}y\left(t\right) \end{array}$$

where −$A_{f}$ is a stable matrix. Substituting y(t) from (14) into (15) gives

(16) $$\begin{array}{c} {\dot{z}}_{f}\left(t\right)=-A_{f}z_{f}\left(t\right)+A_{f}Cx\left(t\right)+A_{f}Ff\left(t\right) \end{array}$$

From (14) and (16), the augmented state-space model with n+p states is achieved as

(17) $$\begin{array}{c} \begin{array}{c} \left[\begin{array}{c} \dot{x}\\ {\dot{z}}_{f} \end{array}\right]=\underset{A_{a}}{\underset{︸}{\left[\begin{array}{cc} A & 0\\ A_{f}C & -A_{f} \end{array}\right]}}\underset{x_{a}}{\underset{︸}{\left[\begin{array}{c} x\\ z_{f} \end{array}\right]}}+\underset{B_{a}}{\underset{︸}{\left[\begin{array}{c} B\\ 0 \end{array}\right]}}u\left(t\right)+\\ \;\;\;\;\;\;\;\;\;\;\;\;\;\;\;\;\;\;\;\;\;\;\;\;\;\;\underset{F_{a}}{\underset{︸}{\left[\begin{array}{c} 0\\ A_{f}F \end{array}\right]}}f\left(t\right)+\underset{D_{a}}{\underset{︸}{\left[\begin{array}{c} D\\ 0 \end{array}\right]}}d\left(t\right)\\ z_{f}\left(t\right)=\underset{C_{a}}{\underset{︸}{\left[\begin{array}{cc} 0 & I_{p} \end{array}\right]}}\left[\begin{array}{c} x\\ z_{f} \end{array}\right] \end{array} \end{array}$$

Equation (17) can be written as

(18) $$\begin{array}{c} \begin{array}{c} {\dot{x}}_{a}\left(t\right)=A_{a}x_{a}\left(t\right)+B_{a}u\left(t\right)+F_{a}f\left(t\right)+D_{a}d\left(t\right)\\ z_{f}\left(t\right)=C_{a}x_{a}\left(t\right) \end{array} \end{array}$$

The output in (18) has formed by the combination of the actual and filtered outputs. It is also assumed that $rank\left(C_{a}F_{a}\right)=rank\left(F_{a}\right)=r$.

To obtain a canonical form for the augmented system (18), a transformation matrix *T* is introduced, so the canonical form is obtained using ${\tilde{x}}_{a}=Tx_{a}$. For this case, the following model is achieved:

(19) $$\begin{array}{c} \begin{array}{c} {\dot{\tilde{x}}}_{a}\left(t\right)={\tilde{A}}_{a}{\tilde{x}}_{a}\left(t\right)+{\tilde{B}}_{a}u\left(t\right)+{\tilde{F}}_{a}f\left(t\right)+{\tilde{D}}_{a}d\left(t\right)\\ z_{f}\left(t\right)={\tilde{C}}_{a}{\tilde{x}}_{a}\left(t\right) \end{array} \end{array}$$

where ${\tilde{A}}_{a}=TA_{a}T^{-1}=\left[\begin{array}{cc} A_{1} & A_{2}\\ A_{3} & A_{4} \end{array}\right]$, ${\tilde{B}}_{a}=TB_{a}$, ${\tilde{F}}_{a}=TF_{a}$, ${\tilde{D}}_{a}=TD_{a}=\left[\begin{array}{c} D_{1}\\ D_{2} \end{array}\right]$ and ${\tilde{C}}_{a}=T^{-1}C_{a}$.

## 3. Sensor Fault Reconstruction Using Second-Order Sliding Mode Observer

A second-order sliding mode observer for (19) is presented as follows:

(20) $$\begin{array}{c} \begin{array}{c} {\dot{\hat{\tilde{x}}}}_{a}\left(t\right)={\tilde{A}}_{a}{\dot{\tilde{x}}}_{a}\left(t\right)+{\tilde{B}}_{a}u\left(t\right)-G_{l}e_{y}\left(t\right)+G_{n}v\left(t\right)\\ \hat{y}\left(t\right)={\tilde{C}}_{a}{\hat{\tilde{x}}}_{a}\left(t\right) \end{array} \end{array}$$

where ${\hat{x}}_{a}$ and $\hat{y}$ denote the estimation of states and outputs, respectively. $G_{l}$ and $G_{n}$ are the observer gains which will be defined later. In (19), v(t) is a nonlinear discontinuous term used to induce the sliding motion.

The output estimation error is defined as

(21) $$\begin{array}{c} e_{y}\left(t\right)=\hat{y}\left(t\right)-z_{f}\left(t\right)={\tilde{C}}_{a}{\hat{x}}_{a}\left(t\right)-{\tilde{C}}_{a}x_{a}\left(t\right)={\tilde{C}}_{a}e_{x}\left(t\right) \end{array}$$

where $e_{x}\left(t\right)={\hat{x}}_{a}-x_{a}$ is the state estimation error. From (18) and (19), one gets

(22) $$\begin{array}{c} {\dot{e}}_{x}={\dot{\hat{\tilde{x}}}}_{a}-{\hat{\tilde{x}}}_{a}=\left({\tilde{A}}_{a}-G_{l}{\tilde{C}}_{a}\right)e_{x}-{\tilde{F}}_{a}f-{\tilde{D}}_{a}d+G_{n}v\left(t\right) \end{array}$$

To force the output estimation error $e_{y}$ to zero in the finite time, the sliding mode surface will be presented as

(23) $$\begin{array}{c} S=\left\{e_{x}\in R^{n+r}:e_{z}=\tilde{C}e_{x}=0\right\} \end{array}$$

The second-order sliding mode output error injection v(t) is presented as follows:

(24) $$\begin{array}{c} \left\{\begin{array}{c} v\left(t\right)=-k_{1}sign\left(e_{y}\left(t\right)\right){\left|e_{y}\left(t\right)\right|}^{0.5}+z\left(t\right)\\ \dot{z}\left(t\right)=-k_{2}sign\left(e_{y}\left(t\right)\right)-k_{3}e_{y}\left(t\right) \end{array}\right. \end{array}$$

where $k_{1}$, $k_{2}$ and $k_{3}$ are the design scalars.

The gain $G_{n}$ is chosen as

(25) $$\begin{array}{c} G_{n}=\left[\begin{array}{c} -L\\ I_{\left(p\times p\right)} \end{array}\right]\in R^{(n+r)\times p} \end{array}$$

where *L* has the following structure:

(26) $$\begin{array}{c} L=\left[\begin{array}{cc} L_{1} & 0_{\left(r\times r\right)} \end{array}\right]\in R^{{}^{(n+r-p)\times p}} \end{array}$$

where $L_{1}\in R^{\left(n+r-p)(p-r\right)}$ is designed such that $\left(L_{1}A_{31}+A_{1}\right)$ is Hurwitz and $A_{31}$ is also the first $\left(p-q\right)$ rows of $A_{3}$.

The observer gain matrix $G_{l}$ is also designed in terms of *L* and a chosen design matrix ${\tilde{A}}_{22}^{s}\in R^{P\times p}$. This chosen matrix can be calculated as the following form [30]:

(27) $$\begin{array}{c} {\tilde{A}}_{22}^{s}=kI_{p} \end{array}$$

The coefficients $k_{1}$, $k_{2}$, $k_{3}$ and *k* are chosen as

(28) $$\begin{array}{c} \begin{array}{c} k>0\\ k_{1}>2\sqrt{\varepsilon }\\ k_{2}>\varepsilon \\ k_{3}>\frac{k(k_{1}^{3}+\frac{5}{4}k_{1}^{2}+\frac{5}{2}\left(k_{2}-\varepsilon \right))}{k_{1}\left(k_{2}-\varepsilon \right)} \end{array} \end{array}$$

where ε is sufficiently large, then $e_{y}\left(t\right)$ converges to the origin in the finite time.

The sensor fault reconstruction signal is presented as

(29) $$\begin{array}{c} \hat{f}=MG_{n}v_{eq} \end{array}$$

where the matrix $M\in R^{p\times l}$ needs to be designed such that $M{\tilde{F}}_{a}=I$. As $e_{y}\left(t\right)$ and $e_{x}\left(t\right)$ converge to zero in the finite time, then, from (22) one obtains

(30) $$\begin{array}{c} 0=-{\tilde{F}}_{a}f-{\tilde{D}}_{a}d+G_{n}v\left(t\right) \end{array}$$

Multiplication of both sides in (30) by *M* implies

(31) $$\begin{array}{c} MG_{n}v\left(t\right)=M{\tilde{F}}_{a}f+M{\tilde{D}}_{a}d \end{array}$$

From (29), one gets:

(32) $$\begin{array}{c} \hat{f}=f+M{\tilde{D}}_{a}d \end{array}$$

Therefore, the effect *d* on the fault reconstruction signal will be minimized if

(33) $$\begin{array}{c} {∥M{\tilde{D}}_{a}∥}_{\infty }<\gamma \end{array}$$

where γ is a small positive scalar.

Let define *P* in the following form:

(34) $$\begin{array}{c} P=\left[\begin{array}{cc} P_{11} & P_{12}\\ P_{12}^{T} & P_{22} \end{array}\right]>0 \end{array}$$

where $P_{11}\in R^{\left(n-p)\times (n-p\right)}$ and $P_{22}\in R^{p\times p}$. Using the Bounded Real Lemma (BRL), the inequality (34) is converted to

(35) $$\begin{array}{c} \begin{array}{c} P=\left[\begin{array}{ccc} \Phi_{11} & \Phi_{12} & -{\left(MA_{3}\right)}^{T}\\ \Phi_{12}^{T} & -\gamma I & MD_{2}^{T}\\ -MA_{3} & WD_{2} & -\gamma I \end{array}\right]<0\\ \left\{\begin{array}{c} \Phi_{11}=P_{11}A_{1}+A_{1}^{T}P_{11}+P_{12}A_{3}+A_{3}^{T}P_{12}^{T}\\ \Phi_{12}=-P_{11}D_{1}+P_{12}D_{2} \end{array}\right. \end{array} \end{array}$$

By obtaining *P* and *M* from (35) and substituting *M* into (32), one obtains

(36) $$\begin{array}{c} \hat{f}≃f \end{array}$$

This concludes the result.

## 4. Simulation Results

To illustrate the accuracy of the results presented in this paper, a state-space model of a heavy-duty diesel engine with initial conditions of $x\left(0\right)={\left[0.1,\;0.5,\;0.51,\;0.1\right]}^{T}$ is obtained as follows:

(37) $$\begin{array}{c} \begin{array}{c} A=\left[\begin{array}{ccc} 3471.35 & 5827.65 & 0\\ 0 & 1467.26 & 0\\ 0 & 0 & 0 \end{array}\right],C=\left[\begin{array}{ccc} 1 & 0 & 0\\ 0 & 0 & 1 \end{array}\right]\\ B=\left[\begin{array}{cc} 0 & 0\\ 173.39 & 0\\ 0 & 3135.39 \end{array}\right],\;D=\left[\begin{array}{c} 1\\ 0\\ 0 \end{array}\right],F=\left[\begin{array}{c} 1\\ 0.5 \end{array}\right] \end{array} \end{array}$$

The value and description of the real system’s parameters are given in Table 1.

By choosing $A_{f}=\left[\begin{array}{cc} 20 & 0\\ 0 & 20 \end{array}\right]$, the following augmented system is obtained:

(38) $$\begin{array}{c} \begin{array}{c} A_{a}=\left[\begin{array}{ccccc} 3471.3 & 5827.6 & 0 & 0 & 0\\ 0 & 1467.3 & 0 & 0 & 0\\ 0 & 0 & 0 & 0 & 0\\ 0.02 & 0 & 0 & -0.02 & 0\\ 0 & 0 & 0.02 & 0 & -0.02 \end{array}\right]\\ B_{a}=\left[\begin{array}{cc} 0 & 0\\ 173.4 & 0\\ 0 & 3135.4\\ 0 & 0\\ 0 & 0 \end{array}\right],D_{a}=\left[\begin{array}{c} 0\\ 1\\ 0\\ 0\\ 0 \end{array}\right],F_{a}=\left[\begin{array}{c} 0\\ 0\\ 0\\ 20\\ 10 \end{array}\right]\\ C_{a}=\left[\begin{array}{cc} 0_{3\times 2} & I_{3} \end{array}\right] \end{array} \end{array}$$

To obtain a canonical form, we define a matrix *T*:

(39) $$\begin{array}{c} T=\left[\begin{array}{ccccc} 1 & 0 & 0 & 0 & 0\\ 0 & 1 & 0 & 0 & 0\\ 0 & 0 & -0.4472 & -0.4000 & 0.8000\\ 0 & 0 & -0.8944 & 0.2000 & -0.4000\\ 0 & 0 & 0 & -0.8944 & -0.4472 \end{array}\right] \end{array}$$

Therefore, the matrices are transformed to

(40) $$\begin{array}{c} \begin{array}{c} {\tilde{A}}_{a}=\left[\begin{array}{ccccc} 1467.3 & 0 & 0 & 0 & 0\\ -5827.6 & 3471.3 & 0 & 0 & 0\\ 0 & -8 & -23.2 & -6.3 & 0\\ 0 & -17.9 & 4 & 8 & -20\\ 0 & 0 & 0.02 & 0 & -0.02 \end{array}\right]\\ {\tilde{B}}_{a}=\left[\begin{array}{cc} -173.4 & 0\\ 0 & 0\\ 0 & -1402.2\\ 0 & -2804.4\\ 0 & 0 \end{array}\right],{\tilde{F}}_{a}=\left[\begin{array}{c} 0\\ 0\\ 0\\ 0\\ -22.3607 \end{array}\right]\\ {\tilde{C}}_{a}=\left[\begin{array}{ccccc} 0 & 0 & -0.4472 & -0.8944 & 0\\ 0 & 0 & -0.400 & 0.200 & -0.8944\\ 0 & 0 & 0.800 & -0.400 & -0.4472 \end{array}\right]\\ {\tilde{D}}_{a}={\left[\begin{array}{ccccc} 0 & 1 & 0 & 0 & 0 \end{array}\right]}^{T} \end{array} \end{array}$$

and the matrices $G_{n}$, $G_{l}$ and *M* are designed as

(41) $$\begin{array}{c} \begin{array}{c} G_{n}=\left[\begin{array}{ccc} -1 & 1 & 0\\ 0 & -2 & 0.5\\ 0.5 & -1 & 0\\ 1 & 0 & 0\\ 0 & 1 & -0.5 \end{array}\right],G_{l}=\left[\begin{array}{ccc} 1 & -1 & 0\\ 0 & -1 & -2\\ 0.5 & -1 & 0\\ 1 & -1 & 0\\ 0 & 0 & 1 \end{array}\right]\\ M=\left[\begin{array}{cccc} 0 & 1 & 2 & -0.044\\ -1 & 0 & 0 & -0.044\\ 0 & 1 & -0.1 & -0.044\\ 0 & 0 & 0 & -0.044 \end{array}\right] \end{array} \end{array}$$

**Figure 2:**
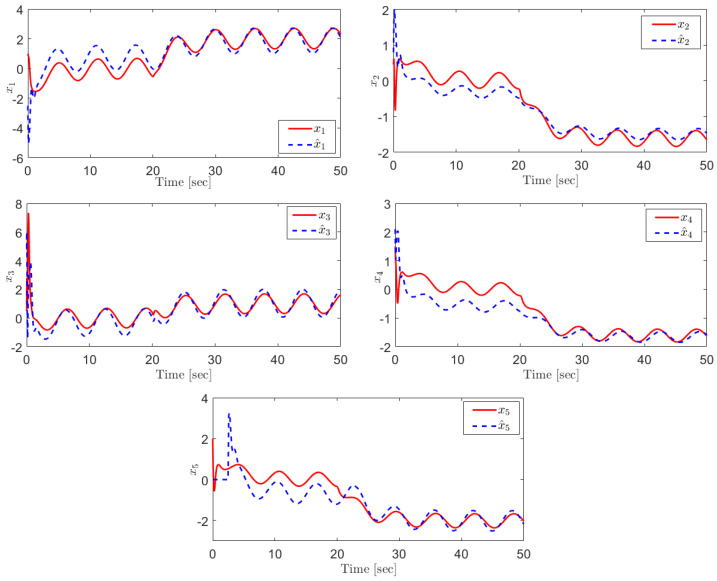
The real states and their estimations.

Choosing ε=4, the observer parameters have been chosen as k=2, $k_{1}=4$, $k_{2}=5$ and $k_{3}=45$. Figure 2 shows the states and their estimation. As can be seen, although there is a disturbance signal d(t)=0.2u(t−20) in the system, each state accurately tracks its estimation signal in the finite time.

For the manifold gas pressure sensor fault, we consider the following fault:

(42) $$\begin{array}{c} f_{P_{im}}=\sin\left(t\right) \end{array}$$

**Figure 3:**
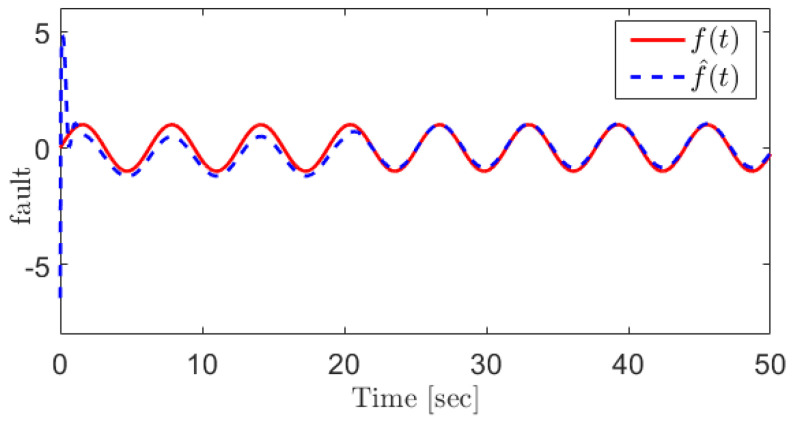
The manifold gas pressure sensor fault and its reconstruction using the proposed SOSMO method.

Figure 3 shows the fault and its reconstruction signal indicating that the sensor faults can be reconstructed using the proposed method.

For the EGR mass flow rate sensor fault, the following fault is assumed:

(43) $$\begin{array}{c} f_{W_{egr}}=u\left(t-10\right)-u\left(t-30\right) \end{array}$$

**Figure 4:**
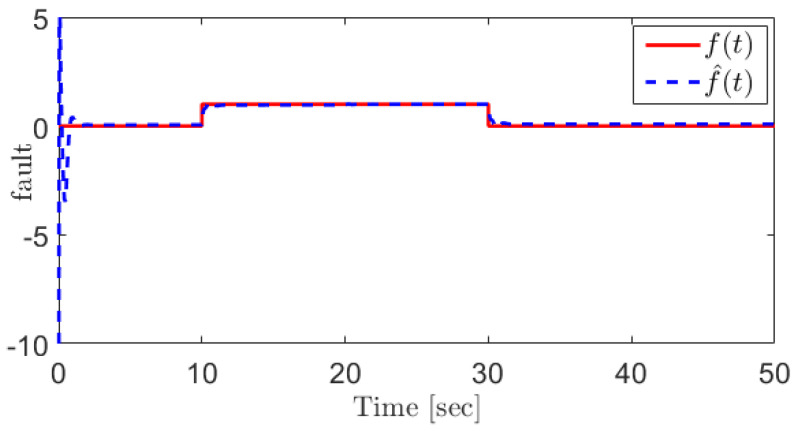
The EGR mass flow rate sensor fault and its reconstruction using the proposed SOSMO method.

**Figure 5:**
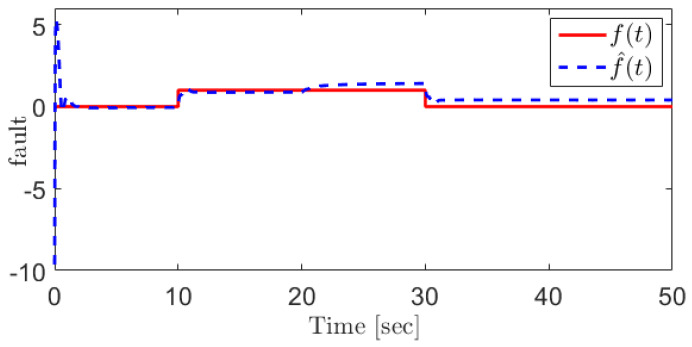
The EGR mass flow rate sensor fault and its reconstruction using SMO method [31].

In this case, the fault can be reconstructed as Figure 4. For the sake of comparison, the sliding mode observer (SMO)-based approach proposed in [31] is applied for the EGR mass flow rate sensor fault reconstruction, and the corresponding result is shown in Figure 5. The results demonstrate that the proposed SOSMO has more robust performance when coping with the unknown disturbances.

Using some quantitative criteria, the performance of the proposed SOSMO method is investigated and compared with the SMO approach. To this aim, the following criteria are defined:

(44) $$\begin{array}{c} \begin{array}{c} J_{observer}=\frac{1}{T_{s}}\int_{0}^{T_{s}}e_{x}^{2}\left(t\right)dt,\;\;\;\;\;e_{x}\left(t\right)=x\left(t\right)-\hat{x}\left(t\right)\\ J_{sensor}=\frac{1}{T_{s}}\int_{0}^{T_{s}}e_{s}^{2}\left(t\right)dt,\;\;\;\;\;e_{s}\left(t\right)=f\left(t\right)-\hat{f}\left(t\right) \end{array} \end{array}$$

where $T_{s}$ denotes the simulation time. Table 2 provides the performance evaluation results for the observer and sensor fault, both for the manifold gas pressure sensor fault and EGR mass flow rate sensor fault. It also shows the result for the EGR mass flow rate sensor fault designed with SMO.

## 5. Conclusions

In this paper, a new robust strategy is proposed for sensor fault reconstruction in a heavy-duty diesel engine in the presence of disturbance. First, a SOSMO was designed using the LMI approach. Then, a sensor fault reconstruction strategy was introduced for the reconstruction of the manifold gas pressure sensor and EGR mass flow rate sensor faults. To verify the advantages of the SOSMO method, the EGR mass flow rate sensor fault reconstruction was compared with a SMO-based method. The results of this comparison were verified both numerically and graphically so that it was proved that the second-order sliding mode observer has more reliable results than the sliding mode observer. According to the results in Table 2, in the SOSMO method, the convergence of the states and the EGR mass flow rate sensor has improved 10.4% and 95.5%, respectively.

## Figures and Tables

**Table 1:** The value and description of the real system’s parameters.

Symbol	Description	Value	Unit
$\eta_{vol}$	Volumetric efficiency	0.043	-
$V_{d}$	Displaced volume	12.4	m${}^{3}$
$\omega_{e}$	Engine speed	1500	$\frac{\mathrm{rad}}{\min}$
$V_{im}$	Intake manifold volume	0.00192	m${}^{3}$
$T_{im}$	Intake gas temperature	315.2	K
$P_{amb}$	The downstream pressure ratio of EGR	$1.55\times 10^{5}$	Pa
$R_{c}$	The radius of the compressor blade	$45\times 10^{-3}$	m
$\varphi_{c}$	Volumetric flow efficiency	0.6	-
$T_{amb}$	Ambient temperature of the intake gas	750	K
$C_{P_{a}}$	Heat capacity of intake gas	1.1	-
$R_{a}$	Ideal gas constant for the air	287	$\frac{J}{\mathrm{kg}\cdot K}$
$\eta_{c}$	Compressor efficiency	0.73	-
$\omega_{t}$	Turbocharger speed	$6.7\times 10^{4}$	$\frac{\mathrm{rad}}{\min}$
$J_{t}$	Rotating inertia of the turbocharger	$75\times 10^{-4}$	kg·m${}^{2}$
$P_{im}$	Intake gas pressure	$1.9\times 10^{5}$	Pa
$\gamma_{a}$	Heat capacity ratio of intake gas	2.2	-
$A_{vgt_{\max}}$	The maximum nominal flow area of VGR	8.5	m${}^{2}$
$P_{em}$	Exhaust pressure before the turbine	$2.25\times 10^{5}$	Pa
$f_{\Pi }$	Mass flow depends on the pressure ratio	0.4	-
$\eta_{tm}$	Turbine efficiency	0.526	-
$C_{pe}$	Heat capacity of exhaust gas	1.31	-
$T_{em}$	Gas exhaust temperature before the turbine	693	K
$R_{e}$	Ideal gas constant for exhaust gas	22.55	$\frac{J}{\mathrm{kg}\cdot K}$
$\gamma_{e}$	Heat capacity ratio of exhaust gas	1.7	-
$A_{egr_{\max}}$	Maximum nominal flow area of EGR	8.4	m${}^{2}$
$\psi_{egr}$	The function of the pressure ratio	1.7	-

**Table 2:** Quantitative performance evaluation.

Sensor Fault	$J_{\mathrm{observer}}$	$J_{\mathrm{sensor}}$
Manifold gas pressure	0.122	0.2572
EGR mass flow rate (SOSMO)	0.112	0.0332
EGR mass flow rate (SMO)	0.125	0.751

## Data Availability

The data that support the findings of this study are available within the article.
